# Comparative examination of the pons and corpus callosum as reference regions for quantitative evaluation in positron emission tomography imaging for Alzheimer’s disease using ^11^C-Pittsburgh Compound-B

**DOI:** 10.1007/s12149-023-01843-y

**Published:** 2023-05-09

**Authors:** Tomohiro Tada, Kazuhiro Hara, Naotoshi Fujita, Yoshinori Ito, Hiroshi Yamaguchi, Reiko Ohdake, Kazuya Kawabata, Aya Ogura, Toshiyasu Kato, Takamasa Yokoi, Michihito Masuda, Shinji Abe, Shinichi Miyao, Shinji Naganawa, Masahisa Katsuno, Hirohisa Watanabe, Gen Sobue, Katsuhiko Kato

**Affiliations:** 1 Department of Radiological Technology, Nagoya University Hospital, 65 Tsurumai-Cho, Showa-Ku, Nagoya, 466-8560 Japan; 2 Department of Neurology, Nagoya University Graduate School of Medicine, 65 Tsurumai-Cho, Showa-Ku, Nagoya, 466-8550 Japan; 3 Department of Radiological and Medical Laboratory Sciences, Nagoya University Graduate School of Medicine, 1-1-20 Daiko-Minami, Higashi-Ku, Nagoya, 461-8673 Japan; 4 Nagoya University Radioisotope Research Center Medical Branch, 65 Tsurumai-Cho, Showa-Ku, Nagoya, 466-8550 Japan; 5 Department of Neurology, Fujita Health University School of Medicine, 1-98 Dengakugakubo, Kutsukake-Cho, Toyoake, Aichi 470-1192 Japan; 6 Department of Neurology, Medical University of Innsbruck, Innrain 52, 6020 Innsbruck, Austria; 7 Department of Neurology, Anjo Kosei Hospital, 28 Higashihirokute Anjo-Cho, Anjo, 446-8602 Japan; 8 Department of Neurology, Toyohashi Municipal Hospital, 50 Hachikennishi, Aotake-Cho, Toyohashi, 441-8570 Japan; 9 Department of Neurology, Okazaki City Hospital, 1-3 Gosyoai, Kouryuji-Cho, Okazaki, 444-8553 Japan; 10 Department of Neurology, Meitetsu Hospital, 2-26-11 Sakou, Nishiku, Nagoya, Japan; 11 Department of Radiology, Nagoya University Graduate School of Medicine, 65 Tsurumai-Cho, Showa-Ku, Nagoya, 466-8560 Japan; 12 Department of Clinical Research Education, Nagoya University Graduate School of Medicine, 65 Tsurumai-Cho, Showa-Ku, Nagoya, 466-8560 Japan; 13 Aichi Medical University, 1-1 Yazakokarimata, Nagakute, Japan; 14 Functional Medical Imaging, Biomedical Imaging Sciences, Division of Advanced Information Health Sciences, Department of Integrated Health Sciences, Nagoya University Graduate School of Medicine, 1-1-20 Daiko-Minami, Higashi-Ku, Nagoya, 461-8673 Japan

**Keywords:** Standardised uptake value ratio, Brain, Positron emission tomography imaging, ^11^C-Pittsburgh Compound-B, Alzheimer’s disease

## Abstract

**Objectives:**

Standardised uptake value ratio (SUVR) is usually obtained by dividing the SUV of the region of interest (ROI) by that of the cerebellar cortex. Cerebellar cortex is not a valid reference in cases where amyloid β deposition or lesions are present. Only few studies have evaluated the use of other regions as references. We compared the validity of the pons and corpus callosum as reference regions for the quantitative evaluation of brain positron emission tomography (PET) using ^11^C-PiB compared to the cerebellar cortex.

**Methods:**

We retrospectively evaluated data from 86 subjects with or without Alzheimer’s disease (AD). All subjects underwent magnetic resonance imaging, PET imaging, and cognitive function testing. For the quantitative analysis, three-dimensional ROIs were automatically placed, and SUV and SUVR were obtained. We compared these values between AD and healthy control (HC) groups.

**Results:**

SUVR data obtained using the pons and corpus callosum as reference regions strongly correlated with that using the cerebellar cortex. The sensitivity and specificity were high when either the pons or corpus callosum was used as the reference region. However, the SUV values of the corpus callosum were different between AD and HC (p < 0.01).

**Conclusions:**

Our data suggest that the pons and corpus callosum might be valid reference regions.

## Introduction

Alzheimer’s disease (AD) is pathologically characterised by neurofibrillary tangles and amyloid β (Aβ) deposition. Aβ deposition is a pathological feature that arises from the earliest stages of AD onset and begins decades before the onset of cognitive decline [1]. ^11^C-Pittsburgh compound-B (PiB) is an amyloid imaging agent developed by Mathis et al., derived from the structure of thioflavin T, which is used to detect Aβ deposition in vitro [2]. Amyloid imaging with ^11^C-PiB enables the visualisation of Aβ deposition in the brain. Amyloid imaging has made it possible to evaluate Aβ deposition in the brain before death; however, it still has to be confirmed at autopsy [3]. Therefore, amyloid imaging using positron emission tomography (PET) is important for the early diagnosis of AD. The development of therapeutic agents for AD has been a focal area of research [4, 5]. Besides helping in AD diagnosis, amyloid imaging with ^11^C-PiB PET also aids in evaluating the therapeutic effect of clinical treatments. Several studies have used the standardised uptake value ratio (SUVR) as a quantitative evaluation of brain PET using ^11^C-PiB [3, 6, 7]. SUVR is obtained by dividing the SUV of the region of interest (ROI) by that of the reference region. Generally, SUVR is evaluated using the cerebellar cortex as the reference region. However, significant amyloid deposition has been reported in the cerebellar cortex of familial AD and in severe AD cases [6]. Furthermore, some patients with cerebral amyloid angiopathy or a certain type of systemic amyloidosis also have significant amyloid deposition in the cerebellum [8, 9]. Moreover, some elderly people have cerebellar disorders, such as cerebellar haemorrhage or cerebellar infarction. Cerebellar infarction accounts for 2–4% of ischaemic strokes, and cerebellar haemorrhage accounts for approximately 10% of all cerebral haemorrhages and, therefore, is not considered a rare disease [10–12]. In these cases, the cerebellar cortex may not be available to use as a reference region. Therefore, few studies have evaluated the pons and the white matter as reference areas [7, 13]. Moreover, the study examined only a small number of subjects or used complicated methods. Thus, in order to improve the generalisability of the conclusions, there is a need for a larger number of study subjects and a less complicated analysis.

In this study, we compared the validity of the pons and corpus callosum as reference regions for the quantitative evaluation of brain PET using ^11^C-PiB with reference to the cerebellar cortex as a reference region. For the initial investigation, we used general AD patients and healthy controls (HCs).

## Materials and methods

### Subjects

In this study, we retrospectively evaluated the data of 86 subjects (23 AD patients and 63 HCs). All subjects underwent magnetic resonance imaging (MRI), PET imaging with ^11^C-PiB, and cognitive function assessments, which included the Mini-Mental State Examination (MMSE) [14], Addenbrooke’s Cognitive Examination Revised (ACE-R) [15], AD Assessment Scale-Cognitive-Japanese (ADAS-cog-j) [16], Logical Memory II of the Wechsler Memory Scale Revised [17], Clinical Dementia Rating (CDR) [18], and CDR Scale Sum of Boxes (CDR-SB) [19].

All AD patients were recruited from the outpatient clinic of our hospital and the Department of Meitetsu Hospital in Nagoya. HCs were recruited from a healthy cohort of an ageing study at our research centre. We defined AD and HCs based on the criteria of Yokoi et al. [20]. In their report, the criteria for diagnosis of AD were as follows: (1) memory complaint; (2) 0.5 or 1.0 in CDR; (3) a score lower than one standard deviation (SD) minus the average of their ages in Logical Memory II; and (4) PiB positive. They assessed the patients as “PiB positive” if the SUVRs, calculated with the cerebellar cortex as a reference region, was larger than 1.5. Clinical diagnoses were made based on the consensus of the three neurologists. This study was approved by the research ethics committee of our hospital (2020-0412, 2019-0033).

### ^11^C-PiB PET imaging

^11^C-PiB PET imaging was obtained at our hospital and performed between 50 and 70 min after an intravenous injection of 555 MBq of ^11^C-PiB. PET imaging was performed using a Biograph16 (Siemens Healthineers, Erlangen, Germany) in the three-dimensional scanning mode, 256 × 256 matrix, and an acquisition time of 20 min. All imaging data were reconstructed by Fourier rebinning and an ordered subset expectation maximisation algorithm, with a combination of numbers of the subset 16 and iteration 2 and a 5 mm Gaussian post-filter using syngo VB40B (Siemens Healthineers, Germany). All imaging data were reconstructed following computed tomography-based attenuation correction and single scatter simulation.

### MR imaging

All MRI scans were performed using MAGNETOM Verio 3 T (Siemens Healthineers, Erlangen, Germany), 32-channel head matrix coil (Siemens Healthineers, Erlangen, Germany) at our research centre. T1-weighted volumetric MR images (repetition time = 2.5 s, echo time = 2.48 ms, flip angle = 8°, field of view = 256 × 256 × 192 voxels, voxel size = 1 × 1 × 1 mm, band width = 170 Hz/pixel, acquisition time = 353 s) were acquired for co-registration with the PET images.

### Image analysis

**Fig. 1:**
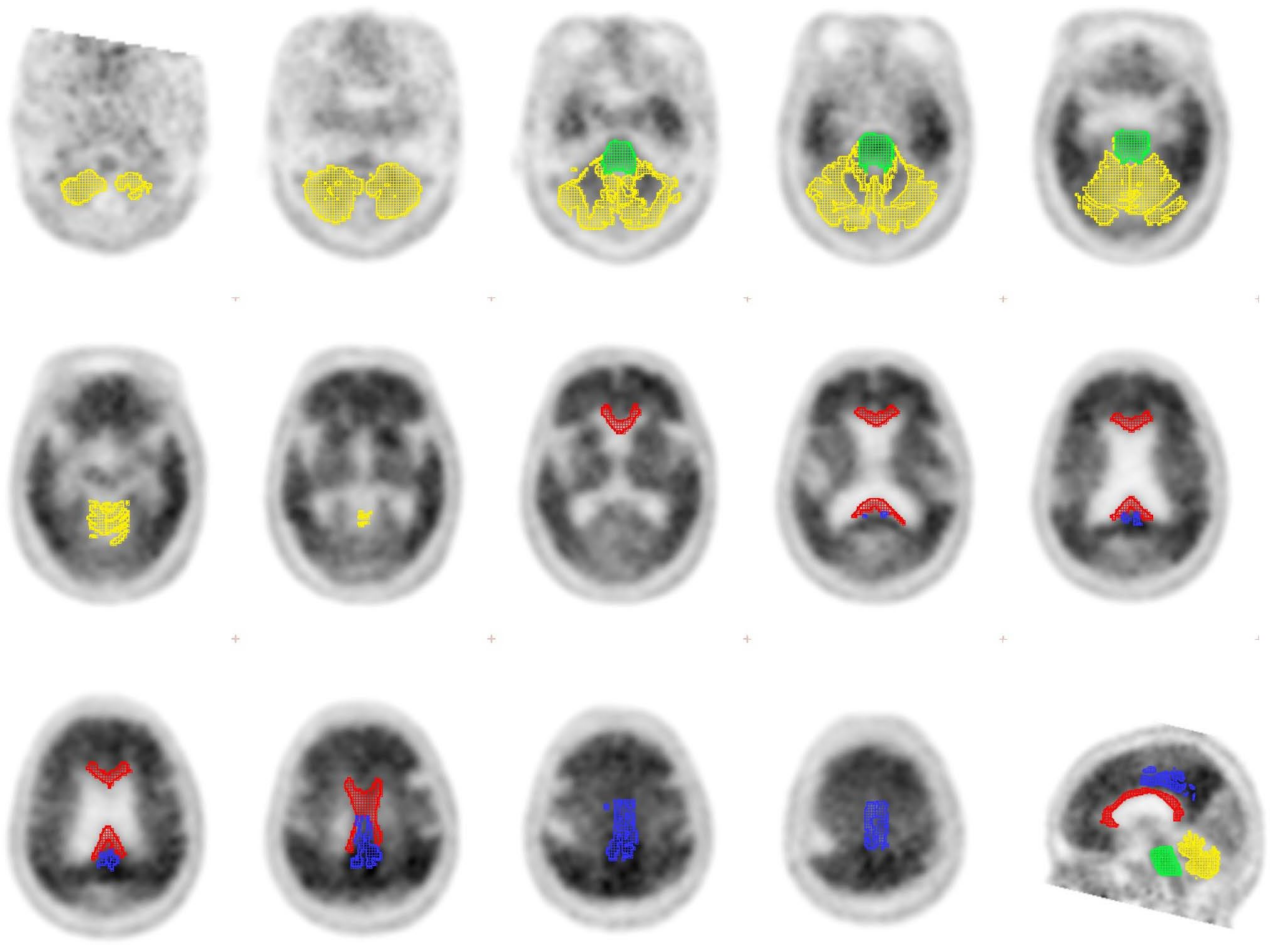
The ROIs of the reference regions. Cerebellar cortex (yellow), pons (green), and corpus callosum (red), posterior cingulate cortex (blue)

We used PMOD software (version 3.9; PMOD Technologies, Zurich, Switzerland) and the PNEURO tool for the quantitative analysis of ^11^C-PiB PET images. Three-dimensional ROIs were automatically placed, and the SUVR of each ROI was obtained using the following method: The T1-weighted volumetric MR images were automatically segmented into gray matter, white matter, and cerebrospinal fluid. ^11^C-PiB PET and segmented MR images for each subject were co-registered, and the MR images were spatially normalised into the standard Montreal Neurological Institute T1 template. The transformation parameters of the normalised MR images were applied to the corresponding PET images. The Hammers N30R83 maximum probability atlas was adapted to the MR images of each subject. ROI information was applied to the PET images of the subject [21, 22]. Figure 1 shows the results of ROIs adapted in the MR images.

It is known that Aβ deposits in the posterior cingulate cortex during the early stages of AD onset [23, 24]. Therefore, SUVR was calculated using the SUVmean of the posterior cingulate cortex as the ROI, and the cerebellar cortex, pons, and corpus callosum as the reference regions. These SUVRs are defined as SUVR_cerebellar cortex, SUVR_pons, and SUVR_corpus callosum.

### Statistical analysis

The results are expressed as mean ± SD. Clinical backgrounds were compared using a non-parametric test (Mann–Whitney U test or chi-squared test). We examined correlations between SUVR_cerebellar cortex, SUVR_pons, and SUVR_corpus callosum using Spearman’s rank correlation coefficient. In addition, the Bland–Altman analysis was performed for comparison between SUVR_cerebellar cortex and both SUVR_pons and SUVR_corpus callosum. We used the Statistical Package for the Social Sciences software (Version 27; SPSS Inc., Chicago, IL, USA) for statistical analyses. We investigated the relationship between SUVR_cerebellar cortex in the posterior cingulate cortex and SUV_pons/SUV_cerebellar cortex and SUV_corpus callosum/SUV_cerebellar cortex. Receiver operating characteristic (ROC) analysis was used to evaluate the sensitivity and specificity of SUVR determined using the cerebellar cortex, pons, and corpus callosum as reference regions. The cut-off values were also calculated. ROC analysis was performed using the statistical software R (version 4.1.3). The SUV mean of pons and corpus callosum were compared between AD and HC. We used the Mann–Whitney U test in the Statistical Package for the Social Sciences software for statistical analyses. The threshold for statistical significance was set at p < 0.01.

## Results

### Patient’s characteristics

There were no significant differences in age at examination, sex, and education levels between AD and HC. However, significant differences in MMSE, ADAS-cog-j, logical memory II, and ACE-R scores were found between AD and HC (Table 1).

**Table 1:** Patient characteristics

	Healthy control (HC)	Alzheimer's disease (AD)	p-value
Number	63	23	N.A
Age at examination	67.4 ± 8.3	68.6 ± 7.6	N.S.^a^
Male:female	22:41	4:19	N.S.^b^
Education (year)^1^	13.9 ± 2.4	13.4 ± 1.8	N.S.^a^
CDR^2^	0.0 ± 0.0	0.67 ± 0.24	< 0.001^a^
CDR-SB^3^	0.0 ± 0.0	2.9 ± 1.1	< 0.001^a^
MMSE^4^	29.3 ± 1.2	23.6 ± 2.7	< 0.001^a^
ADAS-cog-j^5^	3.4 ± 1.7	12.6 ± 5.2	< 0.001^a^
Logical memory II^6^	19.6 ± 6.1	1.0 ± 1.7	< 0.001^a^
ACE-R^7^	97.8 ± 2.4	75.1 ± 8.8	< 0.001^a^

Data are shown as mean ± standard deviation. ^a^HC vs. AD using the Mann–Whitney U test, ^b^HC vs. AD using the chi-square test. *N.S.* not significant, *N.A.* not applicable

^1^Education: Years of school

^2^CDR: Clinical Dementia Rating

^3^CDR-SB: CDR Scale Sum of Boxes

^4^MMSE: Mini Mental State Examination

^5^ADAS-cog-j: AD Assessment Scale-Cognitive-Japanese

^6^Logical memory II: Logical Memory II of the Wechsler Memory Revised

^7^ACE-R: the Addenbrooke’s Cognitive Examination Revised (AC E-R)

### Relationship of SUVR of the posterior cingulate cortex using three reference regions

**Fig. 2:**
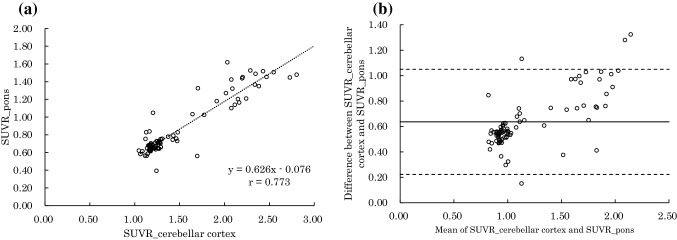
**a** Shows the relationship between SUVR_cerebellar cortex and SUVR_pons (Spearman’s rank correlation coefficient = 0.773). Using the regression equation: SUVR_ pons = 0.626 × SUVR_cerebellar cortex—0.0766. **b** Shows the Bland–Altman plot between SUVR_cerebellar cortex and SUVR_pons. There were fixed (Mann–Whitney U test, p < 0.001) and proportional (Spearman’s rank correlation coefficient = 0.630) biases. Fixed bias was 0.636 (95% confidence intervals [CI] 0.590–0.681)

**Fig. 3:**
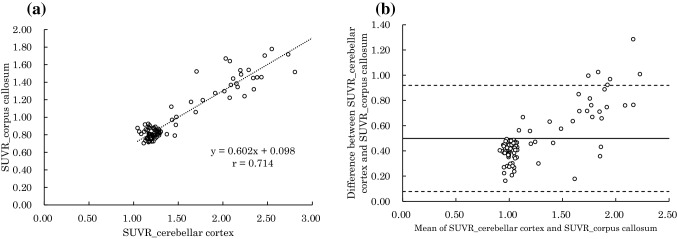
**a** Shows the relationship between SUVR_cerebellar cortex and SUVR_corpus callosum (Spearman’s rank correlation coefficient = 0.714). Using the regression equation: SUVR_corpus callosum = 0.602 × SUVR_cerebellar cortex + 0.098. **b** Shows the Bland–Altman plot between SUVR_cerebellar cortex and SUVR_corpus callosum. There were fixed (Mann–Whitney U test, p < 0.001) and proportional (Spearman’s rank correlation coefficient = 0.796) biases. Fixed bias was 0.499 (CI 0.453–0.545)

The relationship between SUVR_cerebellar cortex and SUVR_pons, and that between SUVR_cerebellar cortex and SUVR_corpus callosum, are shown in Figs. 2 and 3, respectively. Figure 2a shows high correlation between SUVR_cerebellar cortex and SUVR_pons (Spearman’s rank correlation coefficient = 0.773). Using the regression equation, SUVR_pons = 0.626 × SUVR_cerebellar cortex − 0.0766. Figure 2b shows the Bland–Altman plot between SUVR_cerebellar cortex and SUVR_pons. There were fixed bias (Mann−Whitney U test, p < 0.01) and proportional bias (Spearman’s rank correlation coefficient = 0.630. Fixed bias was 0.636 (95% confidence intervals [CI] 0.590−0.681); SUVR_pons was lower than SUVR_cerebellar cortex in the same patient.

Similarly, Fig. 3a shows a high correlation between SUVR_cerebellar cortex and SUVR_corpus callosum (Spearman’s rank correlation coefficient = 0.714). Using the regression equation, SUVR_ corpus callosum = 0.602 × SUVR_ cerebellar cortex + 0.098. Figure 3b shows the Bland–Altman plot between SUVR_cerebellar cortex and SUVR_corpus callosum. There were fixed bias (Mann–Whitney U test, p < 0.01) and proportional bias (Spearman's rank correlation coefficient = 0.796. Fixed bias was 0.499 (95% CI 0.453–0.545); SUVR_corpus callosum was also lower than SUVR_cerebellar cortex in the same patient.

### The relationship of SUVR_cerebellar cortex in the posterior cingulate cortex with SUV_pons/SUV cerebellar cortex and SUV_corpus callosum/SUV cerebellar cortex

**Fig. 4:**
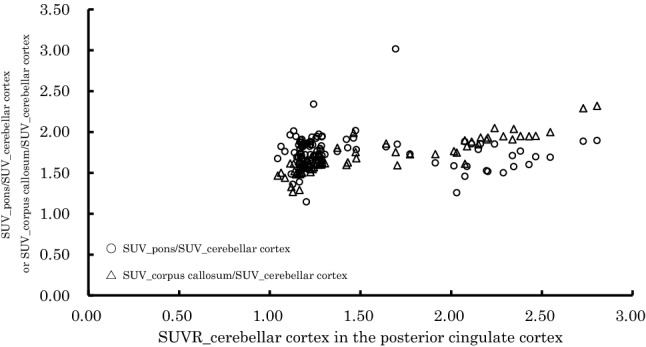
The relationship of SUVR_cerebellar cortex in the posterior cingulate cortex with SUV_pons/SUV cerebellar cortex and SUV_corpus callosum/SUV cerebellar cortex. The SUVR_cerebellar cortex in the posterior cingulate cortex changed; however, the values of SUV_pons/SUV_cerebellar cortex and SUV_corpus callosum/SUVcerebellar cortex are nearly constant between 1.5 and 2.0. That is, the values of SUV_pons and SUV_corpus callosum are approximately 1.5–2.0 times the value of SUV_cerebellar cortex

Figure 4 shows The relationship of SUVR_cerebellar cortex in the posterior cingulate cortex with SUV_pons/SUV cerebellar cortex and SUV_corpus callosum/SUV cerebellar cortex. This figure indicates that the changes in the values of SUVR_cerebellar cortex in the posterior cingulate cortex; however, the values of SUV_pons/SUV_cerebellar cortex and SUV_corpus callosum/SUVcerebellar cortex were nearly constant between 1.5 and 2.0. That is, the values of SUV_pons and SUV_corpus callosum are approximately 1.5–2.0 times the value of SUV_cerebellar cortex.

### Comparison of diagnostic performance due to changes in the reference area

**Fig. 5:**
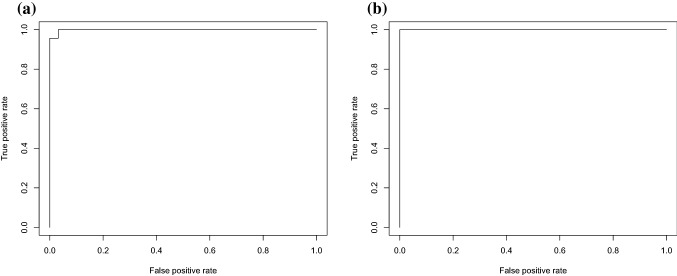
Results of ROC analysis. **a** Shows the result of ROC analysis when the pons was used as the reference region. **b** Shows the result of ROC analysis when the corpus callosum was used as the reference region. When the pons was used as the reference region, sensitivity was 100%; however, specificity was 96.8%. When the corpus callosum was used as the reference region, sensitivity and specificity were both 100%

Table 2 and Fig. 5 show the results of ROC analysis when the pons and corpus callosum were used as the reference region. In this report, all AD were assessed as PiB positive. AD was assessed as PiB positive if the SUVRs, calculated using the cerebellar cortex as the reference region, were larger than 1.5. Accordingly, sensitivity and specificity were both 100% when the cerebellar cortex was used as the reference region. When the pons was used as the reference region, sensitivity was 100%, and specificity was 96.8%. When the corpus callosum was used as the reference region, sensitivity and specificity were both 100%.

**Table 2:** The results of SUVR calculated using SUVmean

	Sensitivity	Specificity	Cut-off
Cerebellar cortex	100% (23/23)	100% (63/63)	1.694
Pons	100% (23/23)	96.8% (61/63)	0.839
Corpus callosum	100% (23/23)	100% (63/63)	1.177

In this study, all AD were PiB positive. They were assessed as “PiB positive” if the SUVRs, calculated with the cerebellar cortex as a reference region, were > 1.5. Therefore, the sensitivity and specificity were 100% when the cerebellar cortex was used as a reference region. The sensitivity was 100%, and the specificity was 96.8% when the pons was used as a reference region. When the corpus callosum was used as a reference region, both the sensitivity and specificity were 100%

### Comparison of ^11^C-PiB accumulation between AD and HC

**Fig. 6:**
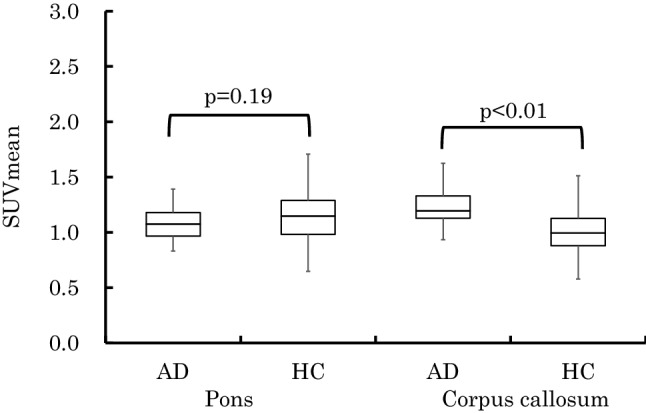
Results of reference regions (SUVmean). There was no significant difference between Alzheimer’s disease (AD) and healthy controls (HCs) in the cerebellar cortex and pons. However, there was a significant difference between AD and HCs in the corpus callosum

A comparison of ^11^C-PiB accumulation between AD and HC in the reference regions is shown in Fig. 6. Mann–Whitney U test revealed that there was no significant difference between AD and HC in the pons (p = 0.25). However, there was a significant difference between AD and HC in the corpus callosum (p < 0.01).

## Discussion

Our data suggested that SUVR_pons and SUVR_corpus callosum were highly correlated with SUVR_cerebellar cortex. When the pons and corpus callosum were used as the reference region, sensitivity and specificity were both high. Therefore, both the pons and corpus callosum might be valid as reference regions for the quantitative evaluation of brain PET using ^11^C-PiB. However, there were fixed and proportional biases when both the pons and corpus callosum were used as reference regions (Figs. 2, 3). Figure 4 shows that the values of SUV_pons and SUV_corpus callosum were nearly constant and are approximately 1.5–2.0 times higher than those of SUV_cerebellar cortex even when the value of SUVR_cerebellar cortex in the posterior cingulate cortex was changed. This is the cause of the systematic error. Therefore, the SUVR value varied depending on the reference region. It is necessary to use the regression equations that were used to perform the measurements in this study to evaluate the SUVR value as well as that of the SUVR_cerebellar cortex.

When the pons was used as the reference region, the specificity was only 96.8% (Fig. 5, Table 2). Edison et al. reported that, due to Aβ deposition in the cerebellar cortex, some AD show no significant differences with HC in cerebral cortical binding using the cerebellar cortex as a reference region, but higher ^11^C-PiB binding in the cerebral and cerebellar cortices was revealed when the pons was used as a reference region [13]. In addition, Yokoi et al. reported that there may have been cases misevaluated as PiB negative when they were PiB positive because of Aβ deposition in the cerebellar cortex.

Although there was no significant difference in the value of SUV between AD and HC in the pons, there was a significant difference in the corpus callosum (p < 0.01) (Fig. 6). The SUVR in AD may be underestimated when the corpus callosum was used as the reference region.

In this study, when the pons and corpus callosum were used as the reference region, SUVR_pons and SUVR_corpus callosum had a high correlation with SUVR_cerebellar cortex. In addition, it was found that even when the pons and corpus callosum are used as reference regions, AD and HC can be discriminated in the same manner as when the cerebellar cortex is used. For these reasons, the pons and corpus callosum are also considered to be valid reference regions for the quantitative evaluation of brain PET in AD patients, using ^11^C-PiB. Therefore, when SUVR cannot be calculated accurately because of Aβ deposition or lesions in the cerebellar cortex, the pons and corpus callosum can be considered valid reference regions.

Moreover, in this study, ^11^C-PiB PET and T1-weighted volumetric MR images were used for quantitative analysis. The T1-weighted MR images were often required to set the ROIs and obtain the SUVR. However, the acquisition of MR images had certain limitations. The acquisition time for the volumetric MR images was approximately 10 min. Patients with metal implants or pacemakers were not eligible for MR scanning. Furthermore, claustrophobic patients had to be excluded. In addition, the software required for setting the ROIs and obtaining SUVR automatically is expensive. An advantage of using the pons and corpus callosum as the reference region is that since their anatomical structures are less complicated than that of the cerebellar cortex, it is possible to set the ROI easily. In particular, using the pons and corpus callosum as the reference region is beneficial when setting the ROI manually.

This study had a few limitations. We did not include subjects whose SUVR could not be calculated accurately due to Aβ deposition in the cerebellar cortex or cerebellar lesions. In future studies, it would be necessary to study subjects in whom Aβ is already deposited in their cerebellar cortex and in patients with cerebellar lesions. We performed the Bland–Altman analysis to compare between SUVR_cerebellar cortex and both SUVR_pons and SUVR_corpus callosum resulting in systematic errors. It is considered that after recalibrating SUVR_pons and SUVR_corpus callosum to set them on the same scale as SUVR_cerebellar cortex would allow for a more sophisticated Bland Altman analysis [25]. In addition, the corpus callosum may have been affected by the partial volume effect because it has a thin structure and is close to the cerebral cortex. The corpus callosum is adjacent to the posterior cingulate cortex. Assuming that the full width at half maximum (FWHM) of the PET is 10 mm, the SUVmean value of the corpus callosum in AD may be overestimated due to the spill over of PiB accumulated in the posterior cingulate cortex. On the other hand, the pons is about 5 cm away from the posterior cingulate cortex. Because it is five times farther away than FWHM, it may not be affected by the spill over of PiB accumulated in the posterior cingulate cortex. It is necessary to examine to what extent the corpus callosum is affected by the partial volume effect due to the spill over from each region in the cerebral cortex.

## Conclusion

We examined the validity of using the pons and corpus callosum as reference regions for the quantitative evaluation of brain PET using ^11^C-PiB, with reference to the cerebellar cortex as the reference region. Our data suggested that SUVR_pons and SUVR_corpus callosum were highly correlated with SUVR_cerebellar cortex. Furthermore, the sensitivity and specificity were high when either the pons or the corpus callosum was used as the reference region. Therefore, the pons and corpus callosum might be valid reference regions.

## Data Availability

The data that support the findings of this study are available from the corresponding author, K.K., upon reasonable request.
